# Supplementary material for: The interactome of intact mitochondria by cross-linking mass spectrometry provides evidence for coexisting respiratory supercomplexes
Source: Mol Cell Proteomics. 2017 Dec 8;17(2):216–32. doi: 10.1074/mcp.RA117.000470 (PMC5795388; doi:10.1074/mcp.RA117.000470)
Supplement: Supplemental Data [file supp_RA117.000470_133922_0_supp_23978_fzffwf.zip › spectra_annotation/mito_spectra_annotation/219-1-8-1-3-1.pdf]

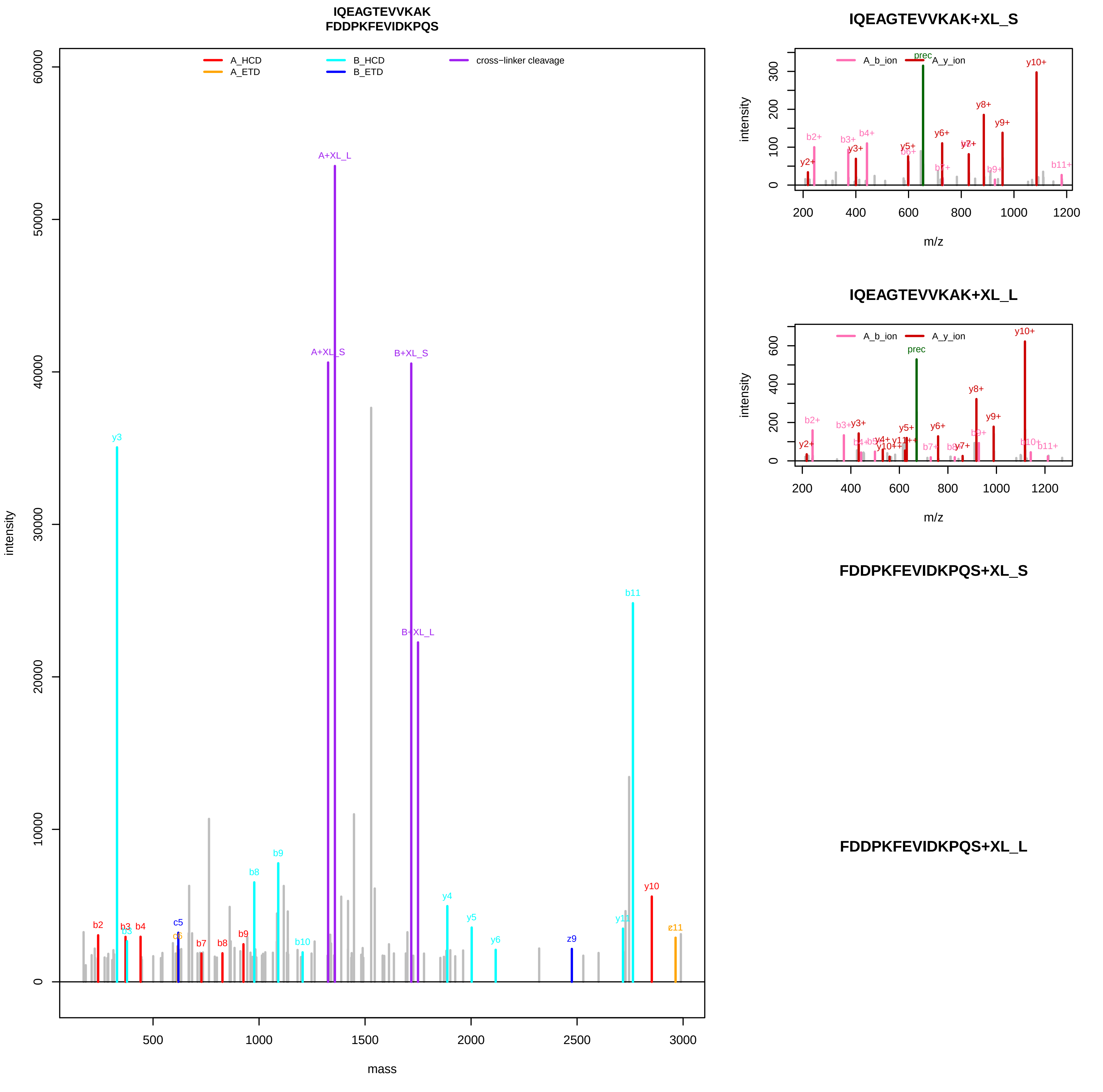
IQEAGTEVVKAK
FDDPKFEVIDKPQS
A_HCD
A_ETD
B_HCD
B_ETD
cross−linker cleavage
A+XL_L
A+XL_S
B+XL_S
B+XL_L
intensity
mass
0
10000
20000
30000
40000
50000
60000
500
1000
1500
2000
2500
3000
y3
b2
b3
b4
c5
c6
b7
b8
b9
b8
b9
b10
y4
y5
y6
z9
y11
b11
y10
z11
IQEAGTEVVKAK+XL_S
A_b_ion
A_y_ion
prec
m/z
IQEAGTEVVKAK+XL_L
FDDPKFEVIDKPQS+XL_S
FDDPKFEVIDKPQS+XL_L
